# Gardner syndrome initially misdiagnosed as bilateral breast malignancy: a case report and literature review

**DOI:** 10.3389/fonc.2026.1691293

**Published:** 2026-02-13

**Authors:** Rong Qian, Guojin Zhang, Juan Liu, Weifang Kong

**Affiliations:** 1 Department of Radiology, Sichuan Provincial People's Hospital, School of Medicine, University of Electronic Science and Technology of China, Chengdu, China; 2 Department of Pathology, Sichuan Provincial People's Hospital, School of Medicine, University of Electronic Science and Technology of China, Chengdu, China

**Keywords:** bilateral breast masses, fibromatosis, Gardner syndrome, imaging findings, literature review, multidisciplinary collaboration

## Abstract

**Purpose:**

To report a rare case of Gardner syndrome (GS) initially presenting with bilateral breast masses, emphasizing the diagnostic challenges of atypical manifestations and the importance of multidisciplinary collaboration.

**Methods:**

A retrospective analysis was conducted using clinical data, multimodal imaging (ultrasound, mammography, magnetic resonance imaging, and computed tomography), histopathological evaluation via core needle biopsy, and multidisciplinary team (MDT) consultation. Colonoscopy was performed to confirm systemic involvement.

**Results:**

A 27-year-old woman presented with bilateral breast masses that were initially suspected to be malignant. Imaging revealed irregular spiculated lesions mimicking invasive carcinoma. Histopathological examination confirmed the presence of fibromatosis in both breasts and the right interpectoral region. Systemic evaluation revealed craniofacial osteomas, subcutaneous/muscular soft tissue tumors, left adrenal adenoma, and multiple colorectal polyps (superficial serrated adenomas). MDT review confirmed GS based on intestinal polyposis, osteomas, soft-tissue tumors, and histopathological criteria.

**Conclusion:**

This case underscores the critical role of multimodal imaging, histopathology, and MDT collaboration in clinical diagnosing GS with rare breast involvement. A systematic evaluation of extracolonic manifestations (e.g., adrenal adenomas) and the recognition of fibromatosis mimicking malignancy are essential for early diagnosis. Genetic evaluation and interdisciplinary management optimize the outcomes of complex hereditary syndromes.

## Introduction

Gardner syndrome (GS), a subtype of familial adenomatous polyposis, is a rare autosomal dominant genetic disorder caused by mutations in the adenomatous polyposis coli (APC) gene. GS is characterized by intestinal and/or extraintestinal manifestations (1, 2). Intestinal features primarily consist of multiple colorectal polyps with high malignant potential, which may progress to colorectal cancer. In contrast, extraintestinal manifestations primarily include osteomas, cutaneous tumors, and soft tissue tumors, which together constitute a classic triad (3). Owing to its prolonged clinical course, diverse presentations, and frequent atypical initial symptoms, GS is prone to misdiagnosis or delayed diagnosis, particularly during the early stages.

This case report presents a retrospective analysis of the clinical diagnostic and therapeutic processes, including imaging findings, in a young female patient with GS who initially presented with bilateral breast masses, an exceptionally rare manifestation. A review of the existing literature is also included to improve the understanding of GS and promote early diagnosis and treatment.

## Case presentation

### History and physical examination

A 27-year-old woman, gravida 2 para 2, presented with a tender right breast mass that she had first palpated 3 years earlier. No associated symptoms such as breast erythema, nipple discharge, or bleeding were observed. The mass gradually enlarged during pregnancy and remained stable after delivery. Six months prior, bilateral breast imaging [mammography and magnetic resonance imaging (MRI)] performed at an external hospital suggested multiple malignant lesions, prompting a referral to our institution. The patient denied any family history of similar conditions or hereditary genetic disorders. She was admitted to the Breast Surgery Department for further evaluation and management under a provisional diagnosis of bilateral breast malignancy.

### Physical examination on admission

Multiple masses with needle-like tenderness were palpated in her right breast. The lesions exhibited regular morphology, ill-defined borders, and moderate mobility, with the largest mass adherent to the chest wall.

Left breast: A firm, irregularly shaped mass with ill-defined borders and moderate mobility was palpated at the superolateral margin.

Lymph nodes: The bilateral axillary lymph nodes were slightly enlarged, with regular morphology, well-defined borders, and moderate mobility.

### Diagnostic workup

#### Breast ultrasound

**Figure 1:**
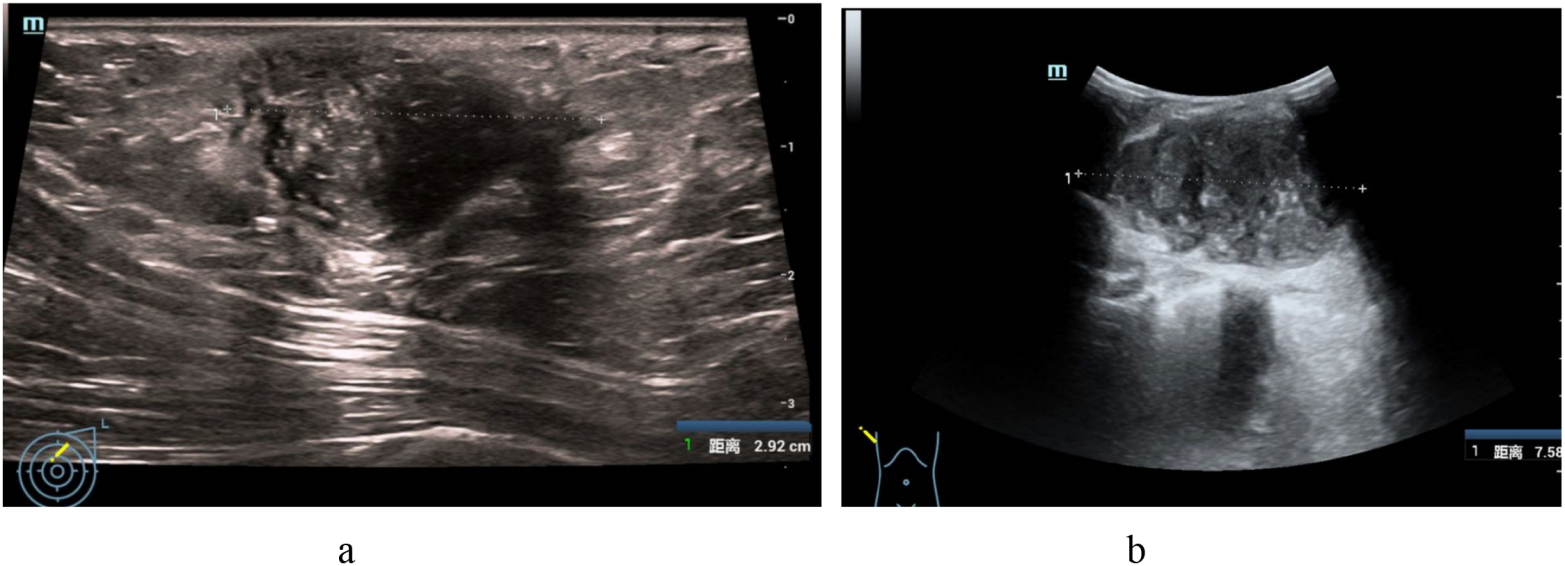
Ultrasound findings. **(a)** Light breast: A hypoechoic mass with relatively well-defined borders, irregular morphology, and heterogeneous internal echogenicity was identified within the fatty layer at the 1 o’clock position, demonstrating intralesional vascularity. **(b)** Right breast: Multiple hypoechoic masses with relatively well-defined borders and moderately irregular morphology were observed in the upper quadrant, exhibiting internal vascularity.

A 55×16×29 mm hypoechoic mass with relatively well-defined borders, irregular morphology, and heterogeneous echotexture was identified in the fatty layer at the 1 o’clock position of the left breast, demonstrating intralesional blood vessels. Multiple hypoechoic masses were observed in the upper quadrant of the right breast, the largest of which measured approximately 107×38×76 mm and 94×35×61 mm. These lesions exhibited relatively well-defined borders, irregular morphologies, and internal vascularity (**Figure 1**).

#### Bilateral mammographic tomosynthesis

**Figure 2:**
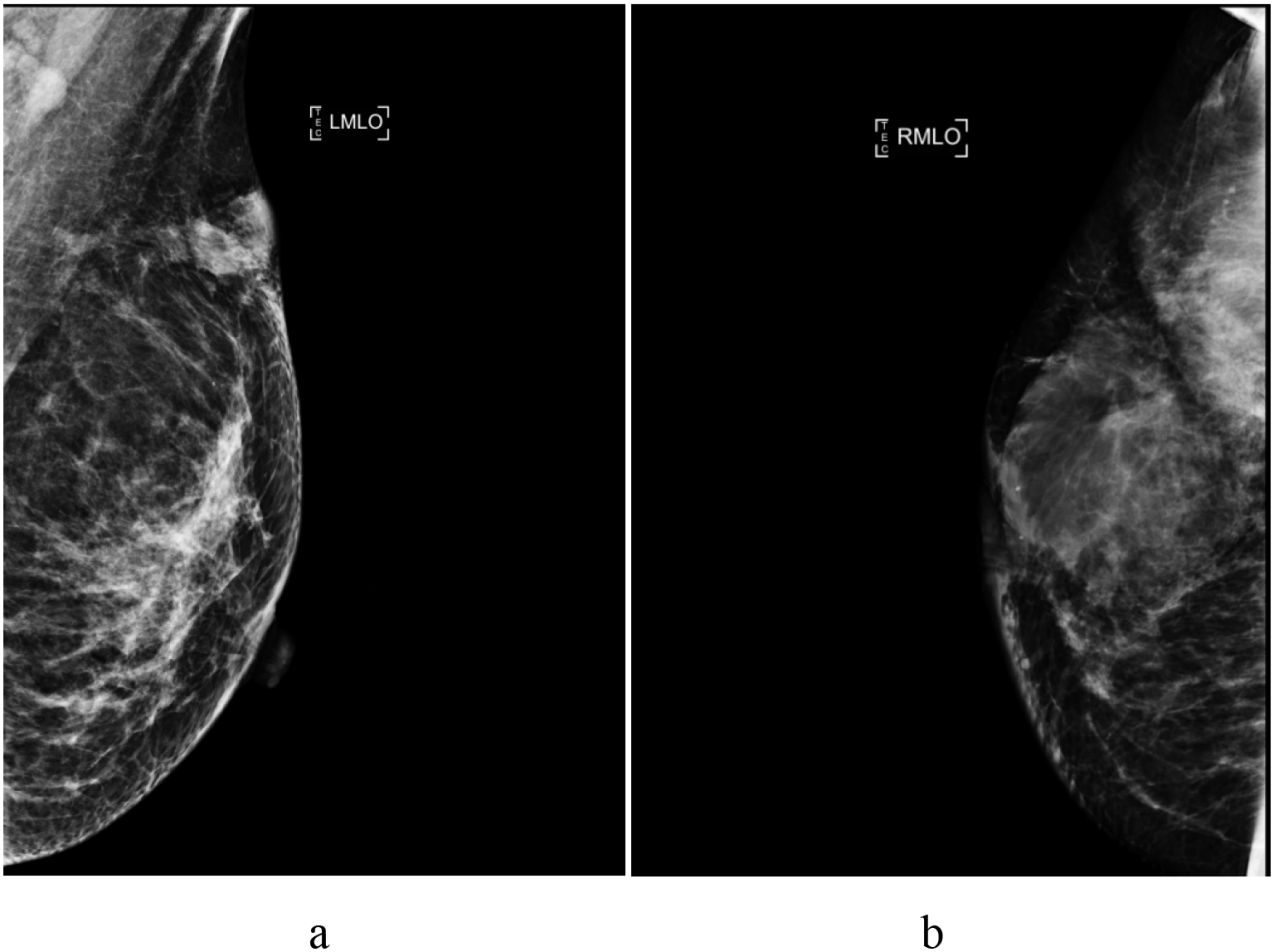
Mammographic findings. **(a)** Left breast: A slightly hyperdense mass with a mildly irregular morphology and ill-defined borders was detected in the superolateral quadrant. **(b)** Right breast: A slightly hyperdense mass with irregular morphology and ill-defined borders was observed in the superolateral quadrant and chest wall.

Ill-defined, slightly hyperdense masses were detected in the superolateral quadrant and chest wall of the right breast and the superolateral quadrant of the left breast, suggesting bilateral breast tumors (BI-RADS category 4 B), which warranted contrast-enhanced MRI (**Figure 2**).

#### Contrast-enhanced breast MRI

**Figure 3:**
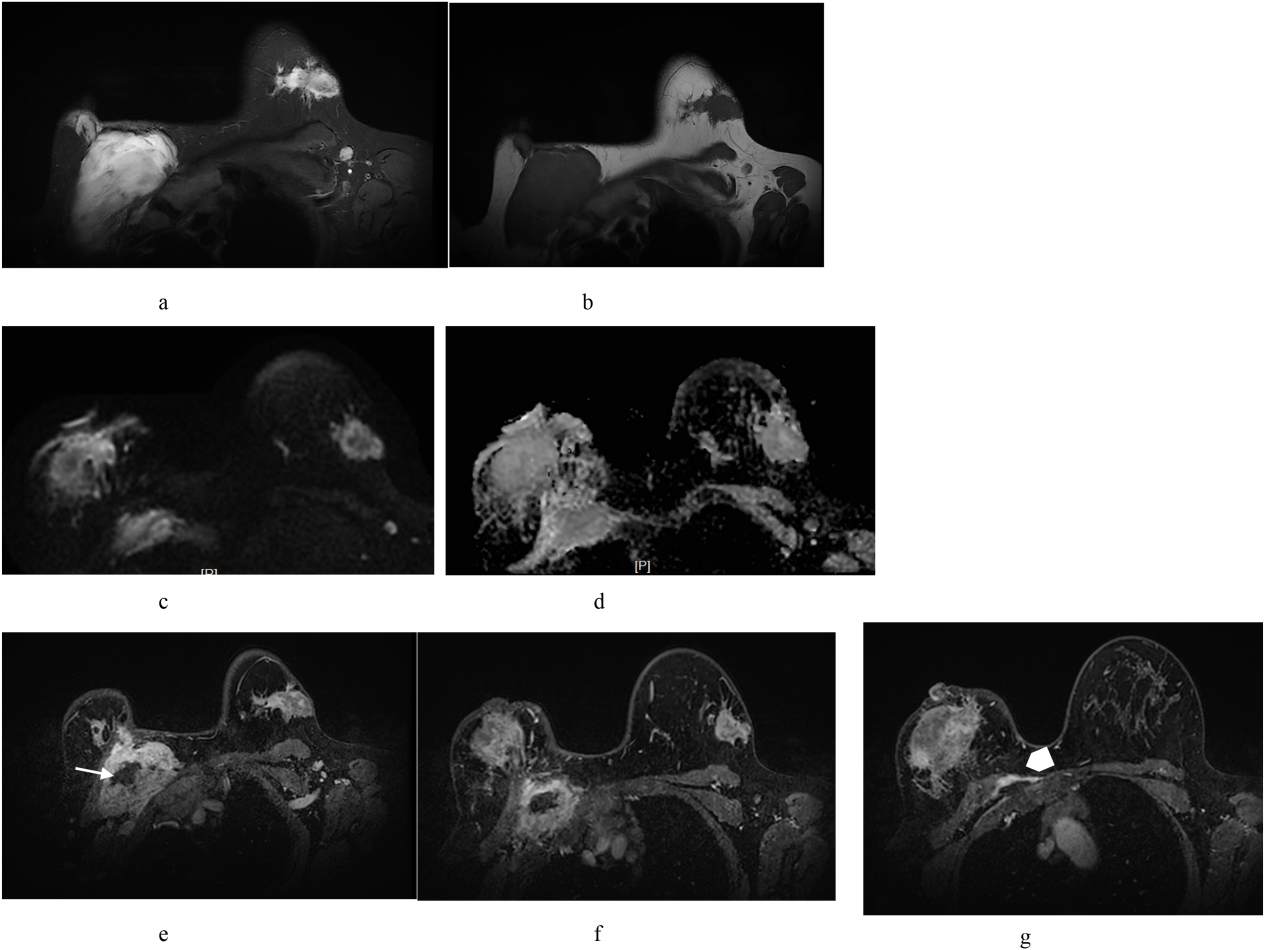
Contrast-enhanced breast magnetic resonance imaging findings. Bilateral breast masses: Irregular lesions with spiculated margins were observed **(a, b)**. Diffusion-weighted imaging (DWI) **(c)** demonstrated restricted diffusion with reduced apparent diffusion coefficient values **(d)**. Post-contrast sequences revealed heterogeneous enhancement **(e-g)**. Right breast mass: Ill-defined borders and chest wall adhesions were observed. A large, irregularly shaped mass with a heterogeneous signal intensity was observed between the right pectoralis major and minor muscles. This lesion exhibited DWI characteristics and enhancement kinetics similar to those of primary breast masses, along with a “band sign” (linear hypointense bands) [**(e)** white arrow] and “fascial tail sign” (fascial extension) [**(f)** white arrowhead] at the interface with adjacent musculature.

Bilateral irregular masses with spiculated margins were identified; the left breast lesion measured 32×29×22 mm, and the right breast lesion measured 84×53×44 mm. Diffusion-weighted imaging (DWI) demonstrated restricted diffusion with reduced apparent diffusion coefficient (ADC) values. Heterogeneous enhancement was observed in post-contrast sequences with type III (washout) time–intensity curve kinetics. The mass in the right breast had ill-defined borders and was adherent to the chest wall. An additional large mass with a heterogeneous signal, irregular morphology, and indistinct margins was observed between the right pectoralis major and minor muscles. This lesion exhibited DWI characteristics and enhancement kinetics similar to those of the primary masses, along with a “band sign” and “fascial tail sign” at the interface with the adjacent musculature. No axillary lymphadenopathy was observed. The imaging findings were consistent with bilateral breast malignancies (BI-RADS category 5) (**Figure 3**). Preoperative computed tomography (CT) evaluation and core needle biopsy of the masses were performed.

#### Contrast-enhanced CT of the head, neck, thorax, and abdomen

**Figure 4:**
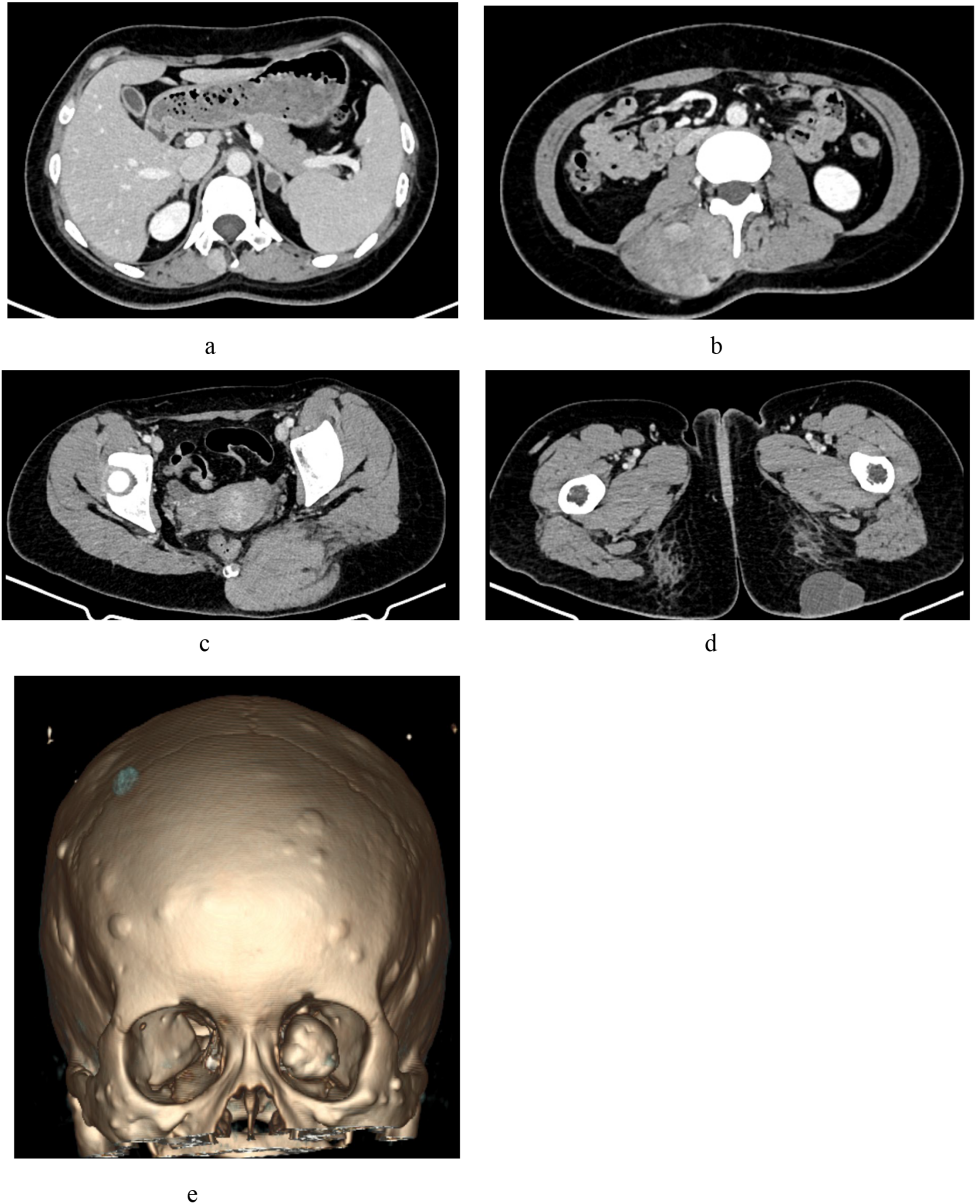
Contrast-enhanced computed tomography findings. **(a)** Left adrenal adenoma: A small, well-defined nodule in the left adrenal gland (maximum diameter: 14 mm). **(b)** Right erector spinae soft tissue mass: A heterogeneously enhancing lesion with ill-defined muscular boundaries. **(c)** Left gluteus maximus soft tissue mass: An irregular, heterogeneously enhancing intramuscular lesion. **(d)** Left gluteal subcutaneous cyst: A non-enhancing, hypodense cystic lesion within the subcutaneous tissue. **(e)** Craniofacial osteomas: Multiple osteogenic lesions involving the left orbital region and cranial bones.

Multiple hyperplastic osteogenic lesions with osteoma formation were observed in the craniofacial bones, most notably in the left mandible. A small soft-tissue nodule was identified on the right frontal area of the scalp. Subcutaneous soft-tissue density masses with partial enhancement and relatively well-defined borders were observed in the thoracic and abdominal walls. Multiple heterogeneously enhanced soft tissue masses with ill-defined margins were detected in the right erector spinae and left gluteus maximus muscles. A small left adrenal adenoma (maximum diameter, 14 mm) was also observed (**Figure 4**).

#### Ultrasound-guided core needle biopsy of bilateral breast and right interpectoral masses

**Figure 5:**
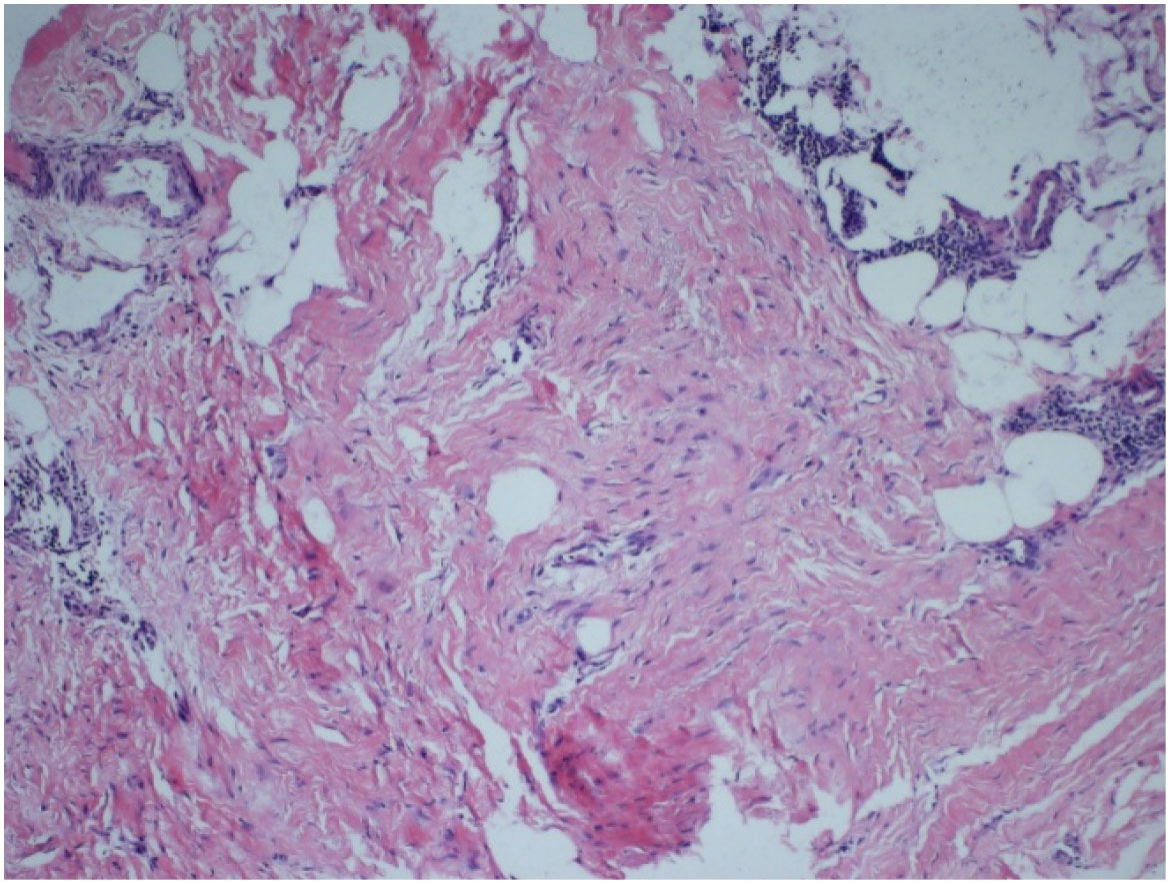
Histopathological findings. Proliferative spindle cell lesions. The immunophenotypic profile supported a fibroblastic/myofibroblastic-derived tumor.

Histopathological examination revealed spindle cell proliferative lesions in the bilateral breast and right interpectoral region, consistent with fibromatosis (**Figure 5**).

### Multidisciplinary consultation

The case was reviewed by a multidisciplinary team (MDT) comprising specialists in breast surgery, radiology, ultrasonography, pathology, oncology, and neurosurgery. Given the presence of widespread soft tissue tumors and craniofacial osteogenic hyperplasia with osteoma formation, along with colonoscopic findings of multiple colorectal polyps (≥100 polyps, biopsy-confirmed superficial serrated adenomas with low-grade glandular intraepithelial neoplasia), GS was diagnosed clinically.

### Final diagnosis

The MDT confirmed the clinical diagnosis of GS based on a comprehensive assessment of clinical, imaging, histopathological, and gastrointestinal findings.

### Post-treatment follow-up and prognosis

The patient underwent endoscopic submucosal dissection for multiple colorectal polyps. She declined further intervention for the soft tissue tumors and osteomas, as well as the associated genetic testing, and was discharged. Follow-up recommendations included regular surveillance colonoscopy and APC gene mutation testing for first-degree relatives. At the 4-month telephone follow-up, the patient reported no significant changes in the breast or other superficial masses, no systemic symptoms, and normal colonoscopy findings in her parents. Neither underwent genetic testing due to personal considerations and financial constraints.

## Discussion

GS is an autosomal dominant disorder, with approximately 20% of cases arising from spontaneous mutations (4). This disease is characterized by the presence of a large number of adenomatous polyps on the intestinal mucosal surface, each with a high potential for malignant transformation. Its extracolonic manifestations primarily include desmoid tumors, osteomas, and epidermoid cysts, among others (5–7). The clinical diagnostic criteria include (1) multiple intestinal polyps (confirmed via colonoscopy or radiography), (2) craniofacial osteomas, (3) soft tissue tumors (e.g., fibromas, epidermoid cysts, lipomas, and leiomyomas), (4) histopathological confirmation, and (5) family history. A clinical diagnosis of complete GS requires the presence of a triad of colorectal polyps, osteomas, and soft tissue tumors, whereas cases lacking one or more of these features are classified as attenuated GS (8). Clinical heterogeneity complicates diagnosis, as some patients exhibit partial manifestations. Cutaneous and skeletal lesions typically precede intestinal polyposis by approximately 10 years of age (9). Osteomas, most commonly involving the jaw and cranial cortex (10–12), and epidermal or sebaceous cysts (13, 14) are hallmark features. Soft tissue tumors frequently arise in the abdominal wall, retroperitoneum, subcutaneous tissues, or muscles, with breast involvement being rare (15, 16).

Breast fibromatosis is a rare neoplasm with poorly understood pathogenesis (15–18). Most cases are sporadic and may be associated with multiple factors, including prior trauma, surgery, silicone implant placement, genetic predisposition, hormonal influences, and pregnancy (19–26). A small proportion of cases are associated with hereditary disorders, such as GS (27). In both sporadic and familial cases, approximately 80% show nuclear expression of β-catenin, a key regulator of tumorigenesis (27–30). Specifically, approximately 20% of patients with sporadic mammary fibromatosis harbor APC mutations (31). These mutations can lead to excessive nuclear accumulation of β-catenin. Elevated levels of β-catenin drive the abnormal activation of the Wnt signaling pathway, which promotes the uncontrolled proliferation and differentiation of fibroblasts, thereby contributing to tumor development (31–34).

Breast fibromatosis commonly originates from the mammary parenchyma or pectoral fascia and often exhibits proximity to the chest wall (35). This condition typically manifests unilaterally; however, bilateral involvement has also been reported (36). It shows a broad age range at onset, with the majority of cases occurring in female patients aged 13 to 80 years (37). Clinically, fibromatosis of the breast typically presents as a solitary, palpable mass characterized by well-defined margins and generally absence of pain. Other common clinical signs include skin and nipple retraction and dimpling (19, 21, 38). Grossly, the tumor is typically firm, pale on the cut surface, and non-encapsulated, with a diameter ranging from 0.3 to 15 cm (39). Histologically, breast fibromatosis is characterized by uniform spindle cells (fibroblasts/myofibroblasts) distributed within the collagenous stroma, arranged in parallel or wavy fascicles, with rare mitotic figures. The tumor exhibits variable architecture, alternating between hypocellular, collagen-rich areas (resembling keloid-like hyalinization) and more cellular regions with a storiform pattern. Its characteristic infiltrative growth pattern manifests as finger-like projections extending into the mammary parenchyma and adipose tissue, reminiscent of phyllodes tumors. Stromal changes may include peripheral lymphocytic infiltration or focal myxoid alterations, similar to those observed in low-grade fibromyxoid sarcoma (40, 41).

Our patient initially presented with bilateral palpable breast masses accompanied by tenderness. Breast ultrasonography, mammographic tomosynthesis, and MRI revealed irregularly shaped, poorly marginated masses. MRI revealed restricted diffusion, heterogeneous enhancement, and type III (washout) kinetic curves, features suggestive of malignancy and closely resembling invasive breast carcinoma. Consequently, the patient was initially misdiagnosed with primary breast malignancy. Notably, the right pectoral soft tissue mass raised diagnostic uncertainty regarding its origin, raising the suspicion of non-epithelial tumors, such as fibromatous or mesenchymal neoplasms. Ultrasound-guided biopsy confirmed the presence of fibromatosis in both breasts and the interpectoral region.

Our patient presented with concurrent fibromatosis in both breasts and the right interpectoral region, characterized by infiltrative growth with indistinct muscular and fascial boundaries. A retrospective analysis of the imaging findings revealed the following results:

- X-ray and ultrasound provided limited discriminative value for fibromatosis.
- MRI features of the bilateral breast masses [irregular morphology, spiculated margins, reduced apparent ADC values, and type III (washout) enhancement kinetics] mimicked those of invasive breast carcinoma. The right breast mass exhibited a “fascial tail sign” at the chest wall interface, suggesting aggressive behavior (35).

### Key MRI Discriminators

The right interpectoral mass displayed heterogeneous signal intensity on non-contrast T1- and T2-weighted sequences, with hypointense areas (“band sign”) and non-enhancing regions on post-contrast imaging. These features correlate with collagenized fibrous tissue during fibromatosis (42), serving as critical differentiators of breast cancer metastasis.

The presence of a chest wall mass with an MRI “band sign” and non-enhancing collagenized components should raise suspicion for fibromatosis, necessitating its inclusion in the differential diagnosis of chest wall lesions.

Following our institutional protocol for suspected primary breast malignancies, the patient underwent contrast-enhanced CT of multiple body regions. Imaging revealed multisystem involvement, including craniofacial osteomas, right frontal scalp and thoracoabdominal wall subcutaneous soft tissue masses, heterogeneously enhancing lesions in the left erector spinae and gluteal muscles, and a left adrenal adenoma. Multisystem diseases present diagnostic and therapeutic challenges. Based on these findings, the MDT at our breast disease center suspected GS. Subsequent colonoscopy identified multiple colorectal polyps (≥100 polyps), which were histologically confirmed as superficial serrated adenomas. The MDT confirmed a clinical diagnosis of GS based on four key criteria: (1) intestinal polyposis, (2) osteomas (predominantly craniofacial), (3) soft tissue tumors (breast, subcutaneous, and muscular), and (4) histopathological confirmation. No definitive family history was observed.

In recent decades, the spectrum of GS-associated extracolonic manifestations has been broadened. In addition to classic osteomas and soft tissue tumors (including fibromatosis), rare comorbidities such as adrenal adenomas, which are typically nonfunctional and incidentally detected on imaging, have been reported. This case involved a non-functional adrenal adenoma, distinct from the exceedingly rare functional adrenocortical tumors previously described in case reports (43–45). Other reported GS-associated lesions include epidermal cysts, dental anomalies, lymphoid hyperplasia, congenital hypertrophy of the retinal pigment epithelium, hepatoblastoma, benign central nervous system tumors, periampullary duodenal cancer, and endocrine neoplasms (thyroid, parathyroid, pituitary, and pancreatic) (9, 43, 46–51). Ophthalmic examination revealed no retinal or fundus abnormalities in our patient.

Atypical presentations and attenuated GS variants pose significant diagnostic challenges, often leading to delayed diagnoses and inappropriate organ-specific interventions. A definitive diagnosis relies on genetic testing for APC mutation (48, 52, 53). According to the American College of Gastroenterology (ACG) and the National Comprehensive Cancer Network (NCCN) guidelines, patients presenting with typical colorectal polyposis and extracolonic manifestations (particularly osteomas and fibromatosis) may receive a clinical diagnosis of Gardner syndrome and should undergo appropriate clinical management, including colonoscopic surveillance and screening for extra-colonic features, even without confirmatory genetic testing. In this case, an accurate clinical diagnosis was achieved through preoperative multimodal imaging (CT/MRI), endoscopic and histopathological evaluation, and MDT collaboration, highlighting the critical role of integrated multidisciplinary approaches in managing complex syndromic disorders.

Despite advances in medical technology, no curative treatment is currently available for GS. Management strategies depend on the lesion location and clinical manifestations. Surgical excision may be considered for osteomas, cutaneous or soft tissue tumors, or fibromatosis that pose cosmetic concerns or cause compressive symptoms. However, given the high recurrence rate of fibromatosis, early resection is no longer recommended. Current guidelines advocate close surveillance for asymptomatic cases, while symptomatic or recurrent fibromatosis may be managed with re-excision, radiotherapy, cryoablation, and/or pharmacotherapy (nonsteroidal anti-inflammatory drugs, hormonal agents, and cytotoxic therapies) (17, 54–56).

GS-associated colorectal polyps have high malignant potential, with up to 80% of patients developing colorectal cancer in adulthood. Regular colonoscopic surveillance and timely polyp removal reduce cancer risk by 55% and improve survival (48, 57, 58). Surgical resection is prioritized for polyps with a confirmed malignancy.

Long-term outcomes depend on early diagnosis, timely intervention, and rigorous follow-up, and the prognosis is influenced by colorectal carcinogenesis and fibromatosis-related morbidities. These factors critically affect patients’ survival and quality of life (48).

## Conclusion

This case report illustrates a rare presentation of GS initially manifesting as bilateral breast masses. A clinical diagnosis was established through comprehensive imaging (CT and MRI), colonoscopy, core needle biopsy, and MDT collaboration.

The take-home message of this case report is as follows:

1. Recognition of atypical clinical and imaging features (e.g., breast fibromatosis mimicking malignancy) is essential for early diagnosis and management.
2. Multidisciplinary teamwork is indispensable for diagnosing complex syndromic disorders and formulating personalized treatment plans.
3. Systematic evaluation of extracolonic manifestations (e.g., adrenal adenomas) expands the diagnostic framework for GS, particularly in cases lacking a clear family history.

This case highlights the importance of integrating multimodal diagnostics, genetic evaluation, and interdisciplinary collaboration to optimize the care of patients with rare hereditary syndromes.

## Data Availability

The original contributions presented in the study are included in the article/Supplementary Material. Further inquiries can be directed to the corresponding author.
